# Effect of curcuminoids and curcumin derivate products on thioredoxin-glutathione reductase from *Taenia crassiceps cysticerci*. Evidence suggesting a curcumin oxidation product as a suitable inhibitor

**DOI:** 10.1371/journal.pone.0220098

**Published:** 2019-07-22

**Authors:** Alberto Guevara-Flores, José de Jesús Martínez-González, Álvaro Miguel Herrera-Juárez, Juan Luis Rendón, Martín González-Andrade, Patricia Victoria Torres Durán, Raúl Guillermo Enríquez-Habib, Irene Patricia del Arenal Mena

**Affiliations:** 1 Departamento de Bioquímica, Facultad de Medicina, Universidad Nacional Autónoma de México, Mexico City, Mexico; 2 Departamento de Química Analítica, Instituto de Química, Universidad Nacional Autónoma de México, Mexico City, Mexico; University of South Carolina, UNITED STATES

## Abstract

Curcuma is a traditional ingredient of some Eastern cuisines, and the spice is heralded for its antitumoral and antiparasitic properties. In this report, we examine the effect of the curcuminoides which include curcumin, demethoxycurcumin (DMC) and bis-demethoxycurcumin (BDMC), as well as curcumin degradation products on thioredoxin glutathione reductase from *Taenia crassiceps* cysticerci Results revealed that both DMC and BDMC were inhibitors of TGR activity in the micromolar concentration range. By contrast, the inhibitory ability of curcumin was a time-dependent process. Kinetic and spectroscopical evidence suggests that an intermediary compound of curcumin oxidation, probably spiroepoxide, is responsible. Preincubation of curcumin in the presence of NADPH, but not glutathione disulfide (GSSG), resulted in the loss of its inhibitory ability, suggesting a reductive stabilizing effect. Similarly, preincubation of curcumin with sulfhydryl compounds fully protected the enzyme from inhibition. Degradation products were tested for their inhibitory potential, and 4-vinylguaiacol was the best inhibitor (IC_50_ = 12.9 μM), followed by feruloylmethane (IC_50_ = 122 μM), vanillin (IC_50_ = 127 μM), and ferulic aldehyde (IC_50_ = 180 μM). The acid derivatives ferulic acid (IC_50_ = 465 μM) and vanillic acid (IC_50_ = 657 μM) were poor inhibitors. On the other hand, results from docking analysis revealed a common binding site on the enzyme for all the compounds, albeit interacting with different amino acid residues. Dissociation constants obtained from the docking were in accord with the inhibitory efficiency of the curcumin degradation products.

## Introduction

Curcuminoids present in the rhizome of turmeric (*Curcuma longa*) include curcumin, the major component (77%), as well as its derivatives, notably demethoxycurcumin (DMC) (17%) and bis-demethoxycurcumin (BDMC) (3%). As turmeric, it is used as a common ingredient in the traditional food of some Asian populations. Curcumin, also called diferuloylmethane (IUPAC name (1*E*, 6*E*)-1,7-bis (4-hydroxy-3-methoxyphenyl)-1,6-heptadiene-3,5-dione) has been subjected to intensive research due to its antioxidative, antitumoral, and antiparasitic properties [1–5]. However, the compound is chemically unstable due to its susceptibility to a variety of chemical modifications, such as oxidation, reduction, conjugation and breakage [6]. Such modifications are dependent on a variety of factors, including the oxygen availability and the interaction of curcumin with solvent, pH, or light [7–9] as well as the presence of other curcuminoids [10]. Particularly importance is the susceptibility of curcumin to spontaneous breakage, which can result in a variety of degradative products [11, 12]. In this sense, it has been reported that the curcumin degradation products feruloyl methane and ferulic acid shows biological effects very similar to that observed with the parent compound [13,14]. As a consequence, some controversy exists about the exact role that curcumin plays in the observed biological effects.

With regard to its potential anti-parasite effects, curcumin has been studied in the Protista *Leishmania major* [15], *Trypanosoma brucei* [16,17], *Giardia lamblia* [18], and *Plasmodium falciparum* [19, 20]; however, the detailed molecular origin of such anti-parasite action has not been thoroughly studied. An inhibitory effect of both curcumin and DMC on thioredoxin reductase (TrxR) activity from *P*. *falciparum* was reported [21]. In this same parasite, one of the three curcuminoids is able to inhibit glutathione reductase (GR). Interestingly, both enzymes showed a higher affinity by DMC [22] as compared with that for curcumin or BDMC. The potential use of curcumin or its derivatives on multicellular parasites, has been poorly investigated.

The flatworm parasites of the Phylum Platyhelminthes, which includes tapeworms (Class Cestoda) and flukes (Class Trematoda) are particularly interesting, as these frequently infect human populations. In these organisms, the classical TrxR and GR enzymes are absent and the thiol-based antioxidative defense system is dependent on a single NADPH-dependent disulfide reductase involved in the reduction of both glutathione disulfide (GSSG) and thioredoxin (Trx) [23–25]. The enzyme, named thioredoxin glutathione reductase (TGR), is an isoform of the high molecular weight variant of TrxR in which a glutaredoxin-like domain has been appended at the N-terminal end of the TrxR module. Like TrxR, TGR is also dependent on an essential selenocysteine residue for enzymatic activity [26]. The disulfide reductase activity of the enzyme is critical for parasite survival, as has been demonstrated in *Schistosoma mansoni* [27], *Taenia crassiceps* [24], and *Echinococcus granulosus* [23]. Although the presence of TGR in mammals has been demonstrated, its expression is restricted to the spermatid stage of sperm maturation [28]. Hence, TGR represents a potential target for drug-based anti-helminth therapy.

The inhibitory effect of curcumin on the activity of some enzymes is well documented [29–31]; however in many cases the purity of the compound used in the activity assays was not reported [32–34]. Furthermore, the possibility that the observed inhibitory effect was due either to autoxidation or degradation products of curcumin was not explored. In the present work we studied the effect of curcumin, DMC, BDMC and six of its degradation products on *Tc*TGR activity *in vitro*.

## Materials and methods

### Chemicals and enzyme

The curcumin degradation products feruloylmethane (FM), vanillin (V), vanillinic acid (VA), ferulic aldehyde (F-Ad), and 4-vinylguaiacol (4VG), as well as the curcuminoids curcumin (65% purity), demethoxycurcumin (DMC) and bis-demethoxycurcumin (BDMC) were obtained from Sigma-Aldrich (St. Louis Mo, USA) both with ≥ 98% purity. PMSF, albumin, EDTA, Tris, NADPH, glutathione oxidized (GSSG), reduced glutathione (GSH), *N*-Acetyl-*L*-cysteine (NAC), DEAE-cellulose, and HA-Ultrogel were also obtained from Sigma-Aldrich with the highest purity degree. 2’5’-ADP Sepharose^™^ 4B was from GE Healthcare Bio-Sciences AB. Ferulic acid (FA) was purchased from Santa Cruz Biotechnology, Inc. 1,4-Dithio-*DL*-threitol (DTT) was from Fluka, Chemika-BioChemika. Curcumin with purity over 98% (pure curcumin) was a gift of Dr. Raúl Enriquez (Instituto de Química, UNAM, México). Curcumin 5 mM was dissolved in DMSO and stored at room temperature. Under these conditions, the compound was demonstrated to be stable as evidenced by essentially constant absorbance at 426 nm after 15 days of storage. Thioredoxin glutathione reductase (EC 1.8.1.B1) from *T*. *crassiceps* (*Tc*TGR) was purified as described previously [35] with minor modifications [36]. The protein concentration of the purified enzyme was determined spectrophotometrically by reading the absorbance at 460 nm (ɛ = 11.3 mM^-1^cm^-1^) due to its FAD content.

### Spectrophotometrical analysis

The absorption spectrum of curcuminoids (curcumin, DMC and BDMC) and the different curcumin degradation products (CDP) FM, V, VA, F-Ad, 4VG, and FA were recorded in a UV/Vis Beckman DU 730 Spectrophotometer. All the compounds were dissolved in 100% DMSO at a concentration of 5 mM. For the spectral analysis, each compound was diluted from its corresponding stock solution to a final concentration of 50 μM in 0.1 M Tris/HCl (pH 7.8) containing 1 mM EDTA (TE buffer). The effect of NADPH and/or GSSG on the stability of the curcuminoids was analyzed by following the absorbance at 426 nm immediately after the addition of 100 μM NADPH and/or 70 μM GSSG to the corresponding compound in TE buffer.

The detection of the curcumin oxidation products (COP) was performed by following the changes in the absorption spectrum immediately after a 100-fold dilution of the curcumin stock solution in TE buffer.

### GSSG-reductase activity of TGR

TGR activity was monitored spectrophotometrically by following the decrease in absorbance at 340 nm (ɛ = 6.22 mM^-1^cm^-1^) due to its NADPH-dependent ability to reduce GSSG [35, 36]. To determine the inhibitory ability of either curcuminoids, curcumin degradation products or curcumin oxidation products we used the following protocols: *a) Curcuminoids*. An enzyme aliquot (30 μL) was incubated in the presence of 1 μM NADPH; then, a 1.5 μL aliquot of the above mixture was added to 50 μM of the corresponding curcuminoid solution in TE buffer. At different incubation times, a small aliquot containing both NADPH and GSSG was added to start the reaction in a total volume of 600 μL. The enzyme activity of a reaction mixture prepared in the absence of any curcuminoid was taken as 100% relative activity *b) Curcumin degradation products (CDP)*. The enzyme was treated as above and 1.5 μL aliquot of the mixture was added to the corresponding CDP solution at a selected concentration followed by a 2 min incubation period. Then, both NADPH and GSSG were added to start the reaction. The enzyme activity of a reaction mixture prepared in the absence of any CDP was taken as 100% relative activity. *c) Curcumin oxidation products (COP)*. Curcumin final concentration 50 μM was incubated 15 min in TE buffer. Then, both TGR and the substrates NADPH and GSSG were added to start the reaction. Final concentrations of enzyme, NADPH and GSSG were 13 nM, 100 μM and 70 μM respectively. A unit of enzyme activity is defined as the amount of protein needed to oxidize one μmol NADPH per minute at 25°C.

### Homology modeling of thioredoxin glutathione reductase (TGR) of *T*. *solium*

The nucleotide sequence of TGR of *Taenia solium* deposited in parasitic genome database GeneDB (GeneDB ID TsM_000506200) (http://www.genedb.org) was used for homology modeling. The mature protein predicted from this cDNA sequence is 623 amino acids. Using the program Basic Local Alignment Search Tool (BLAST; https://blast.ncbi.nlm.nih.gov/Blast.cgi) [37] and database of the Protein Data Bank; the structural model was built based on the thioredoxin glutathione reductase (*Eg*TGR) of *Echinococcus granulosus* (PDB: 5W1L) [38], which revealed a coverage of 98% and the highest sequence identity (90%) with the target. Multiple alignments were performed using the Constraint-based Multiple Alignment Tool (COBALT; https://www.ncbi.nlm.nih.gov/tools/cobalt/re_cobalt.cgi) servers. Subsequently, using MODELLER 9.17 r10881 with the multiple-model protocol, 1000 *T*. *solium*-TGR models were constructed. Later, a simple structural refinement of a full atom was performed using the "relax" application of Rosetta. The final model was called *Ts*TGR and validated using the Verify-3D (structure evaluation software) [39] and Whatcheck (protein verification tools software) [40] computer programs.

### Docking protocol

Docking was conducted using the *Ts*TGR model and ligands (Curcumin, DMC, BDMC, spiroepoxide, 4VG, VA, and FA). The structure of ligands were constructed using HyperChem 8 software. All structures of the ligands were minimized using Gaussian 09, revision A.02 (Gaussian Inc., Wallingford, CT) at DTF B3LYP/3-21G level of theory. The structures of *Ts*TGR and ligands were further prepared using the utilities implemented by AutoDockTools 1.5.4 (http://mgltools.scripps.edu/). All hydrogen atoms as well the Kollman united-atom partial charges were added to the protein structures, while Gasteiger-Marsili charges and rotatable groups were assigned automatically to the structures of the ligands, and the active torsions were added to the structures of the ligands. Blind docking was carried out with AutoDock4 version 4.2 (http://autodock.scripps.edu/) [41, 42] using the following conditions: default parameters for the Lamarckian genetic algorithm with local search, number of individuals in population (150), maximum number of energy evaluations (2.5 million), maximum number of generations (27000), rate of gene mutation (0.02), rate of crossover (0.8), and 2000 runs for docking. Electrostatic grid maps were generated for each atom type using the auxiliary program AutoGrid4 that is part of the software AutoDock4. The initial grid box size was 60Å × 60Å × 60Å in the x, y, and z dimensions. Docking was analyzed with AutoDockTools using cluster analysis, Friesner [43] and PyMOL Delano [44] software.

### Statistics

All enzyme assays were performed in triplicate. Results are expressed as mean ± standard deviation.

## Results

### TGR activity in the presence of curcuminoids

**Fig 1:**
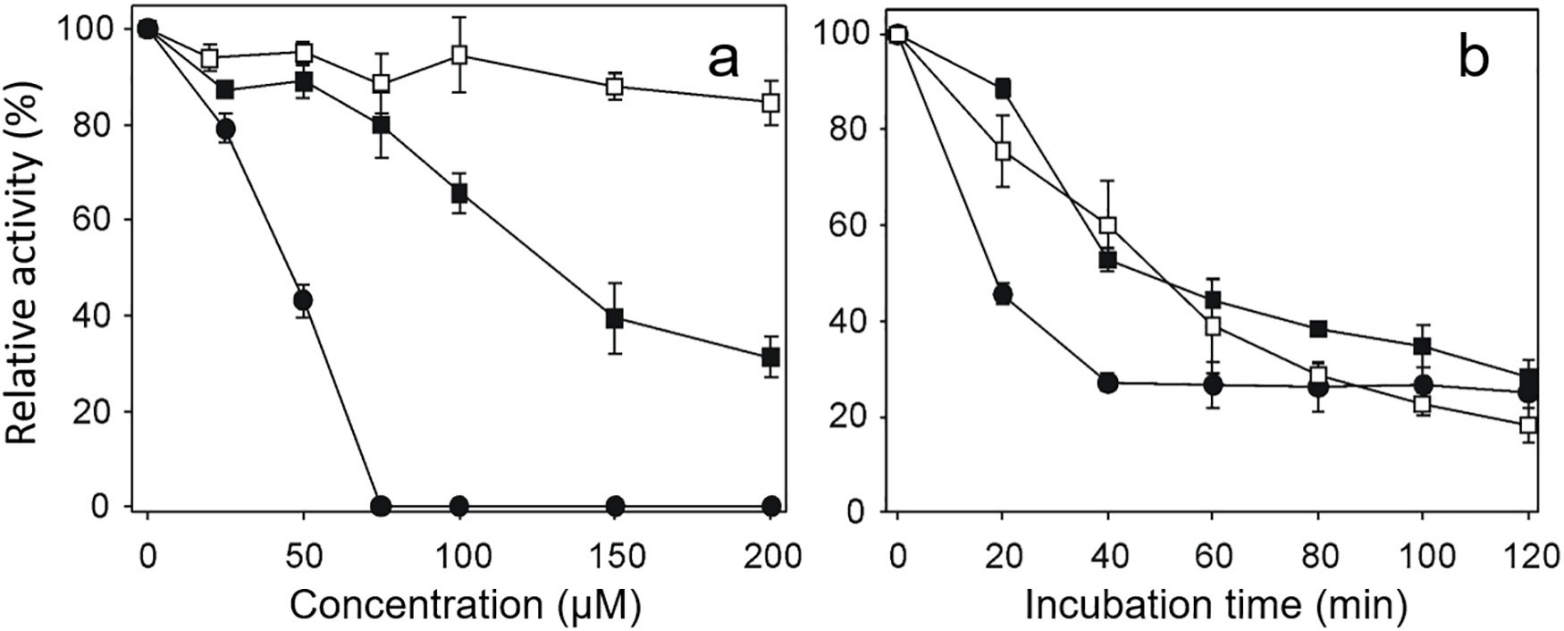
The inhibitory ability of curcuminoids on the GSSG reductase activity of *Tc*TGR. (a). A reduced enzyme aliquot was incubated in TE buffer in the presence of either Curcumin (□), DMC (■), or BDMC (●) at the indicated concentration. After 3 min, both NADPH and GSSG were added to start the reaction. (b). An enzyme aliquot was incubated with 50 μM of the corresponding curcuminoid as described above. At the indicated times, the reaction was started by adding both NADPH and GSSG. For both experiments, the final concentrations of TGR, NADPH and GSSG were 13 nM, 100 μM and 67 μM, respectively. The decrease at 340 nm preceding the addition of GSSG was subtracted in each assay.

Curcumin, DMC, and BDMC inhibited GSSG reductase activity of *Tc*TGR, with a maximal inhibition of 10% for curcumin, 65% inhibition for DMC, and 100% apparent inhibition for BDMC at 200 μM of the compound (Fig 1a), see below.

The presence of 50 μM of either curcumin, DMC or BDMC in the reaction mixtures resulted in inhibition of the GSSG reductase activity of TGR (Fig 1b). However, it is worth noting the long incubation times required for a significant degree of inhibition. Thus, after 40 min incubation with either curcumin or DMC, a 60% residual activity was still present. Under the same conditions, 25% residual activity was obtained with BDMC, and after 40 min incubation its inhibition was constant. The other two compounds, the residual activity of about 20% was observed after 120 min incubation. Longer incubation times were not analyzed. The long incubation times needed to observe a significant inhibition of the enzyme activity is consistent with the results reported for the inhibition of recombinant TrxR by curcumin [32].

### NADPH stabilizes curcumin

#### Effect of the reaction components on curcuminoid stability

**Fig 2:**
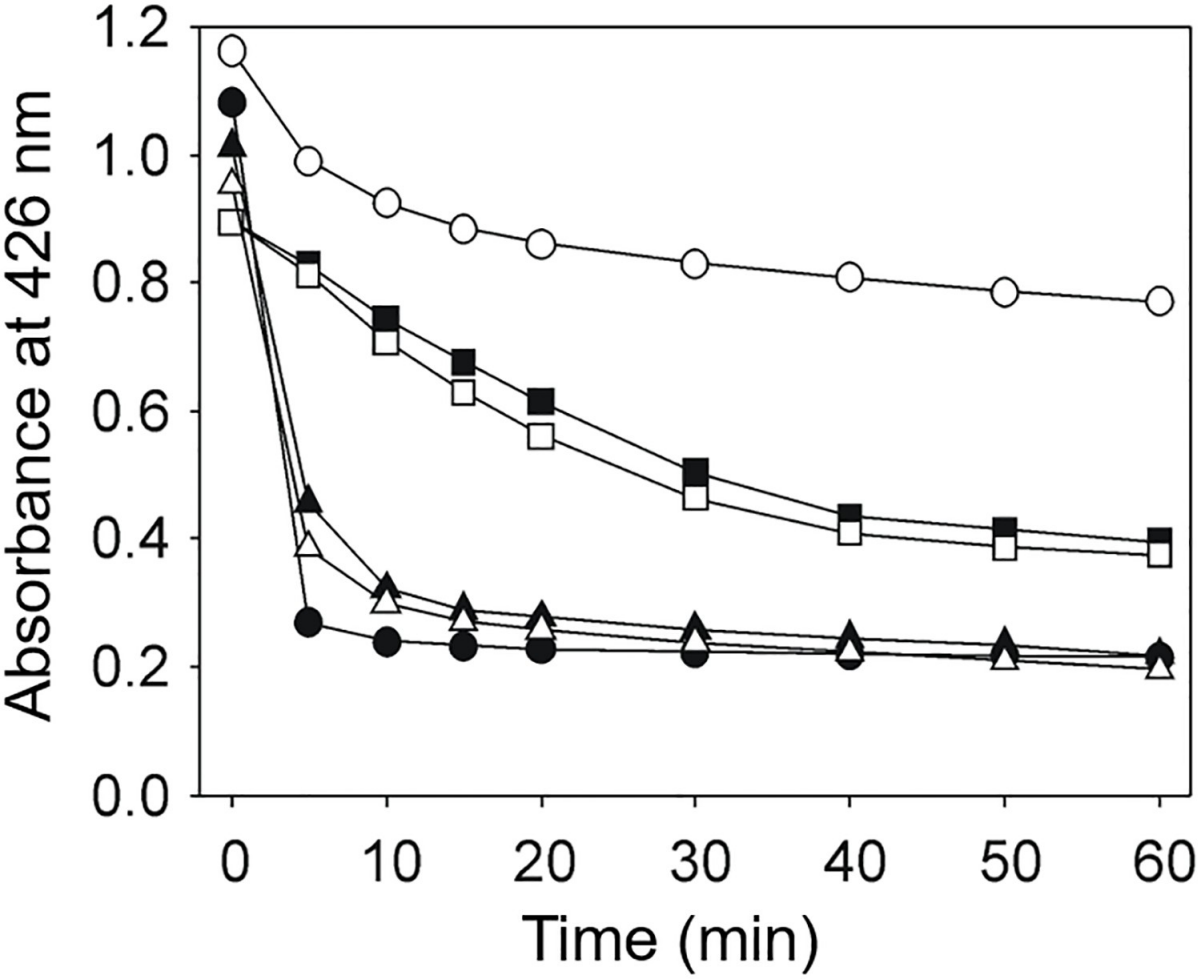
Effect of NADPH on curcuminoid stability. A sample of either curcumin (circles), DMC (squares), or BDMC (triangles) was diluted in TE buffer to a final concentration of 50 μM either in the absence (black symbols) or in the presence (white symbols) of 100 μM NADPH. At the indicated times, the absorbance at 426 nm was determined.

Based on all the available evidence concerning the instability of curcumin in aqueous solutions [7], it was necessary to analyze the potential effect that the components of the reaction mixture could play on curcumin stability. NADPH demonstrated a significant stabilizing effect on the absorbance of the curcumin as a function of time (Fig 2). After 30 min incubation in the presence of the reduced nicotinamide nucleotide, barely a 25% diminution in the absorbance at 426 nm was observed. By contrast, in the absence of NADPH, the drop in the absorbance was as great as 82%, interestingly, no effect was observed with NADP^+^, strongly suggesting that the stabilization of curcumin by NADPH was due to a reducing effect. With regards to the related DMC and BDMC, the presence of NADPH in the incubation mixture had no effect on the stability of the two curcuminoids. On the other hand, the presence of the substrate GSSG, either in the presence or absence of NADPH, had no stabilizer effect on the absorbance of the curcuminoids, even after one hour incubation.

#### NADPH protects curcumin from degradation

**Fig 3:**
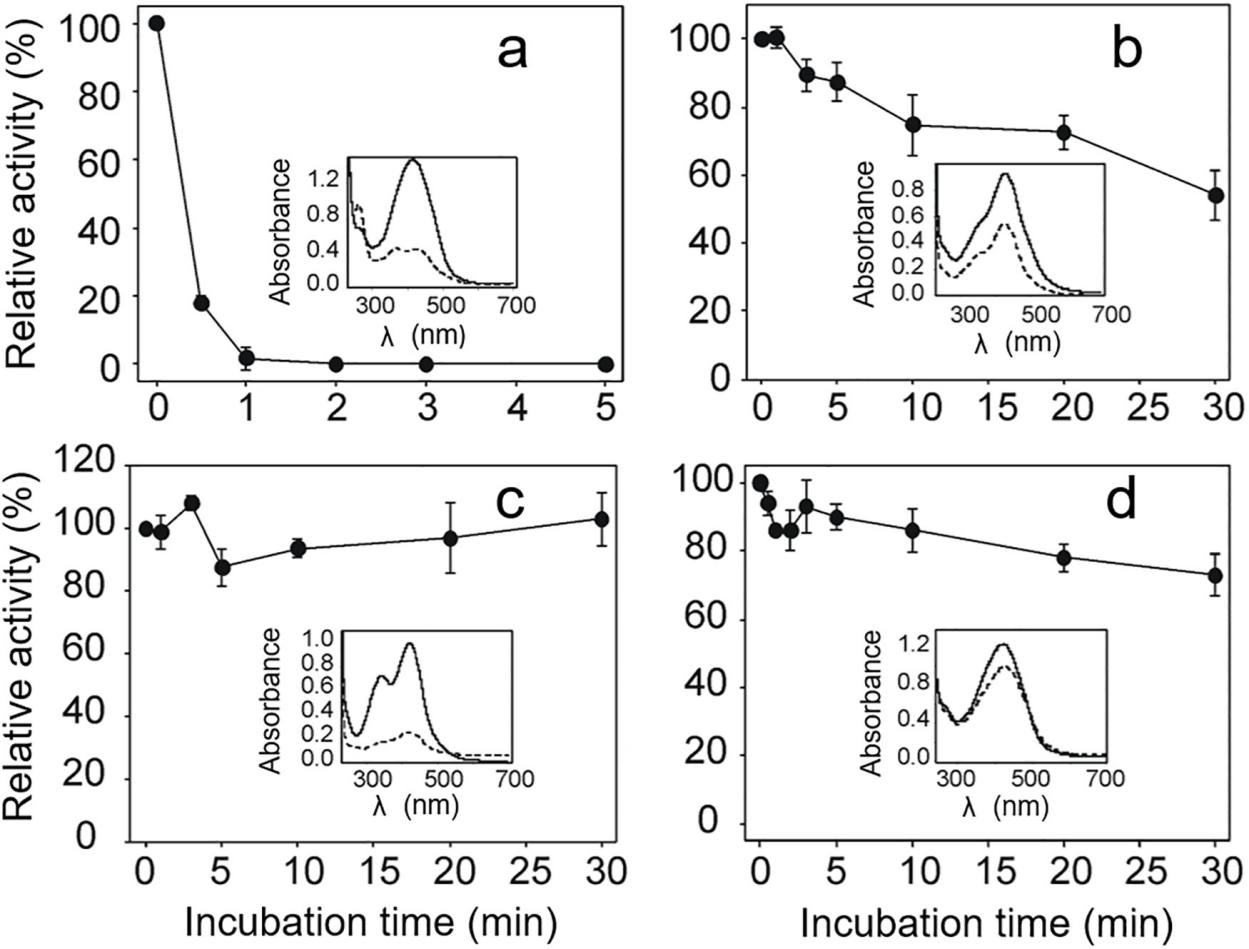
Effect of long-term incubation of curcuminoids on *Tc*TGR inhibition. Curcuminoid samples were diluted in TE buffer at 50 μM final concentration. After 30 min dilution, an enzyme aliquot was added and incubated at the indicated times with TGR. Then, NADPH was added to obtain the baseline for two min and the reaction was started by adding GSSG. (a) Pure curcumin; (b) DMC; (c) BDMC; (d) impure curcumin. Inset in each panel shows the absorption spectra of the corresponding curcuminoid immediately (continuous line) and 30 min after dilution (long dashed line).

In order to gain insight into the origin of the inhibitory ability of the curcuminoids, the following experiment was carried out. A sample of the corresponding curcuminoid compound was diluted in TE buffer to a final concentration of 50 μM. The absorption spectrum was recorded immediately as well as 30 min after dilution. At this latter time, a small enzyme aliquot was added to the corresponding curcuminoid solution, and at different incubation times, both NADPH and GSSG were added to start the reaction. Dilution of curcumin (Fig 3a inset) resulted in significant changes both in intensity and in the shape of the profile of its UV/visible absorption spectrum. These changes are consistent with previous reports [45] and have been associated with an autoxidative process of curcumin in aqueous solutions, resulting in the formation of derivative compounds. Thus, the absorbance bands with maxima at 263 and 360 nm, were assigned to the spiroepoxide and vinylether derivatives, respectively. By contrast, dilution of the related curcuminoids DMC and BDMC resulted only in a decrease in absorption intensity (Fig 3b and 3c inset), while retaining the shape of the profile. These observations strongly suggest that the decrease in absorption intensity for both the DMC and BDMC diluted solutions are due to a precipitation phenomenon.

A solution of diluted curcumin result in an effective inhibition on TGR activity such that after 1 min incubation with the enzyme no GSSG reductase activity was detected (Fig 3a). This result is in radical contrast with the inhibition experiment for curcumin and also for the BDMC inhibition shown in Fig 1. Thus, after 30 min incubation of TGR with the BDMC diluted solution, no inhibition was observed. In contrast, the inhibition degree observed by DMC was essentially identical to that reported in Fig 1.

The above-described experiment was also carried out with an impure curcumin preparation (65% purity), in which both DMC and BDMC are present. After 30 min dilution in TE buffer, only a minor decrease in absorbance was observed (Fig 3d inset), and barely 20% inhibition of the enzyme activity was detected after 30 min incubation with the diluted impure curcumin (Fig 3d).

### Simultaneous curcumin oxidation and TGR inhibition

**Fig 4:**
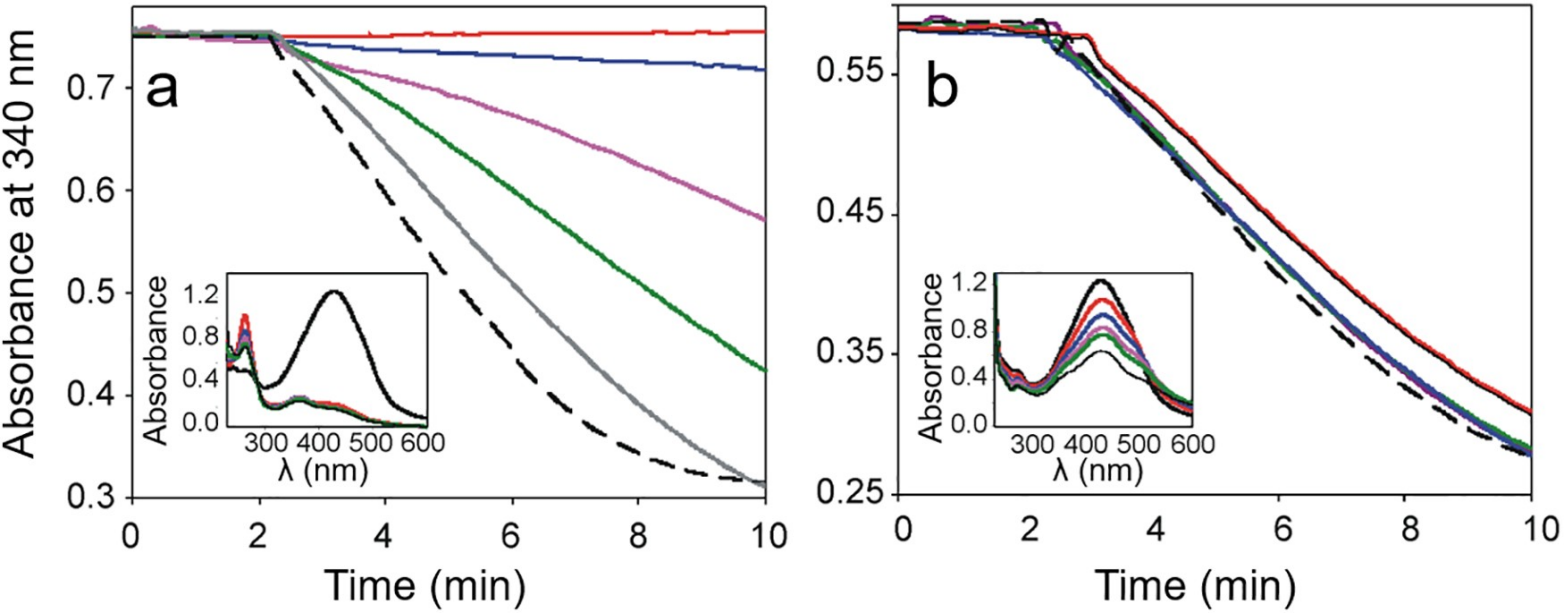
Temporary inhibition of *Tc*TGR by a curcumin oxidation product (COP). A curcumin sample was diluted in TE buffer to a final concentration of 50 μM either in the absence (a) or in the presence (b) of 100 μM NADPH. At selected time points both the enzyme activity and the absorption spectra (inset) were determined. For the former, an enzyme aliquot containing both enzyme and NADPH was added to the curcumin solution and incubated for two min; then, GSSG was added to start the reaction. The colors code for the selected times were as follows: 15 min (red); 45 min (blue); 75 min (pink); 120 min (green); 240 min (grey). Control line is shown as a black dashed line.

**Fig 5:**
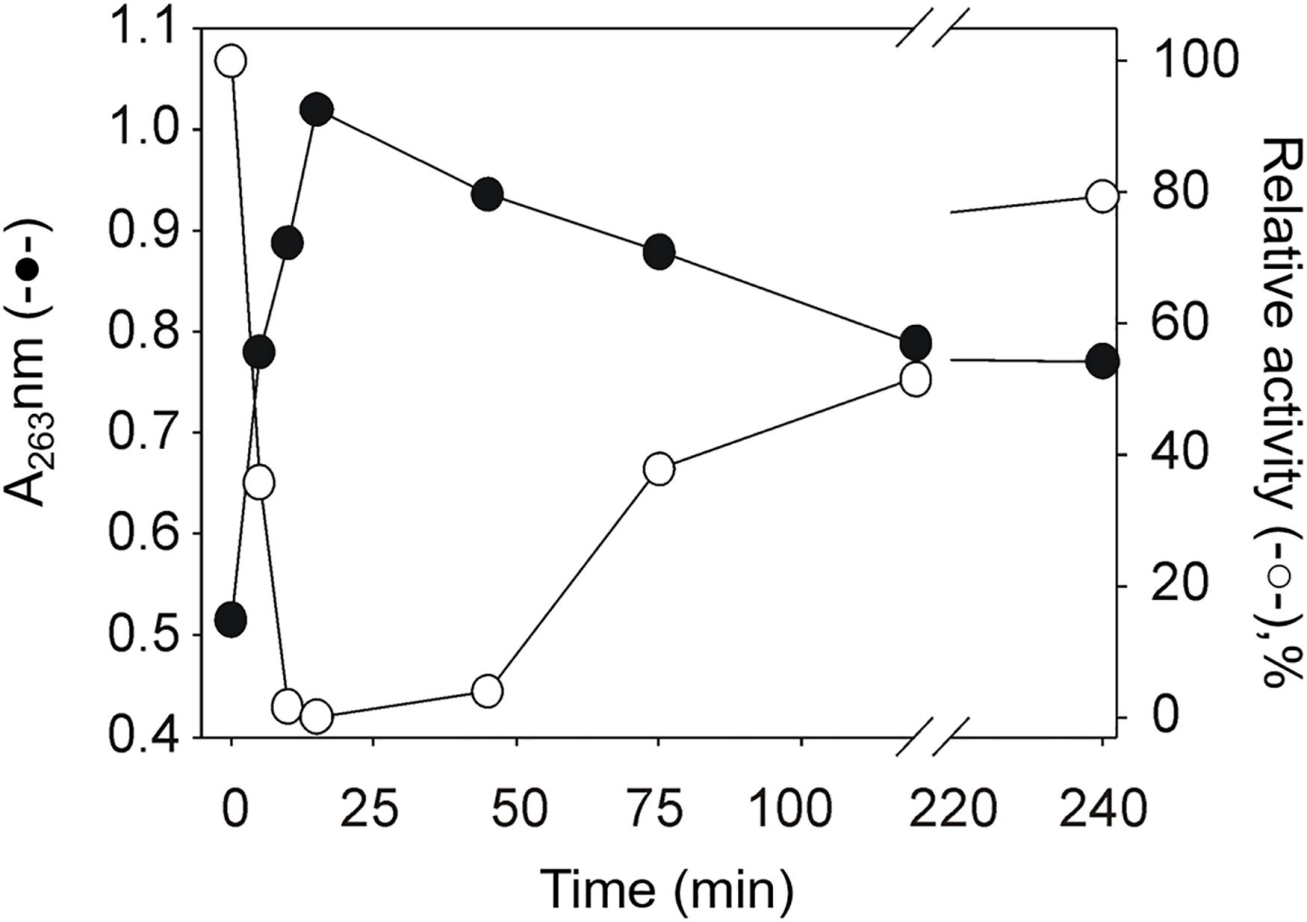
Time dependence of relative activity and absorbance at 263 nm. The absorbance at 263 nm (●) and the relative activity (○) are plotted as a function of time after dilution of a curcumin sample in TE buffer. Data were taken from the experiments showed in panels (a), and (b) of Fig 4 except for the points that correspond to 5 and 10 min.

The appearance of the absorption peak with a maximum at 263 nm has been associated with the formation of the spiroepoxide derivative during curcumin autoxidation [45]. To test the possibility that the effective inhibition of enzyme activity by curcumin (Fig 3a) was correlated with the formation of this putative curcumin derivative, the following experiment was performed. A sample of curcumin was diluted in TE buffer either in the absence (Fig 4a) or in the presence of 100 μM NADPH (Fig 4b). At selected time intervals, an aliquot was obtained and mixed with TGR and substrates to determine the enzyme activity. When pure curcumin was diluted in the absence of NADPH, the maximal inhibitory ability was reached after 15 min dilution (Fig 4a red line), concomitant with the maximal value of absorbance at 263 nm (Fig 4a inset). When pure curcumin was diluted in the presence of NADPH (Fig 4b), no inhibition of the enzyme activity was observed. Under these conditions, the absorbance band centered at 263 nm was not detected in the absorption spectrum (Fig 4b inset). However, intriguingly, in the assay of samples of longer TGR incubation times the inhibition decreases indicating the presence of a compound with a short lifetime reaching 80% of the control activity after 4 h incubation (Fig 4a grey line). A correlation between the maximum of enzyme inhibition and the maximal absorbance at 263 nm is clearly evident within 15 minutes of reaction (Fig 5). These results suggest that the inhibitory ability of pure curcumin depends on the appearance of a compound with the short half-time suggested (Fig 4a), whose formation is avoided in the presence of NADPH.

#### TGR activity in the presence of curcumin oxidation products (COP) complexed with thiols and effect of thiols on the activity of TGR inhibited by COP

It has been demonstrated that in the presence of thiol compounds (e.g., GSH or NAC) curcumin oxidation products are able to adduct covalently, with the spiroepoxide intermediate of curcumin autoxidation being the main derivative product [46]. To test the possibility that such compounds could protect against TGR inhibition, its effect on TGR inhibition was analyzed. As shown in Table 1, pre-incubation of COP with an equimolar concentration of either GSH, DTT, or NAC effectively protects TGR from inhibition (A). With NAC, full protection of the enzyme activity was reached at 1 mM of the reductant. By contrast, when the corresponding thiol was added to the inhibited enzyme by COP, the activity is recovered to a lesser degree (B). These results indicate that these reducer compounds interact with oxidized curcumin, thus preventing the inhibition of the TGR. These data suggest that through SH groups.

**Table 1:** Effect of thiols on COP and on the TGR inhibition. COP was used as an inhibitor of TGR activity (see Materials and methods). In both experiments, the control represents 100% of TGR activity.

*A*		*B*	
Reductant (50 μM)	Activity (%)*	Reductant (50 μM)	Recovered activity (%)*
COP + GSH	105.0 ± 1.5	+ GSH	14.7 ± 1.0
COP + DTT	98.0 ± 2.9	+ DTT	20.3 ± 1.1
COP + NAC	53.8 ± 5.1	+ NAC	18.1 ± 3.0
COP + NAC (1 mM)	101.3 ± 2.8		

*A*. COP was incubated with either GSH, DTT or NAC for 1 min and then TGR, NADPH, and GSSG were added and activity was measured.

*B*. TGR was incubated with COP for 5 min (8% residual activity); then, the corresponding thiol compound at the indicated concentration was added and the reductase activity measured.

* Results are presented as means ± S.D. of three independent experiments.

#### TGR activity in the presence of curcumin degradation products (CDP)

**Fig 6:**
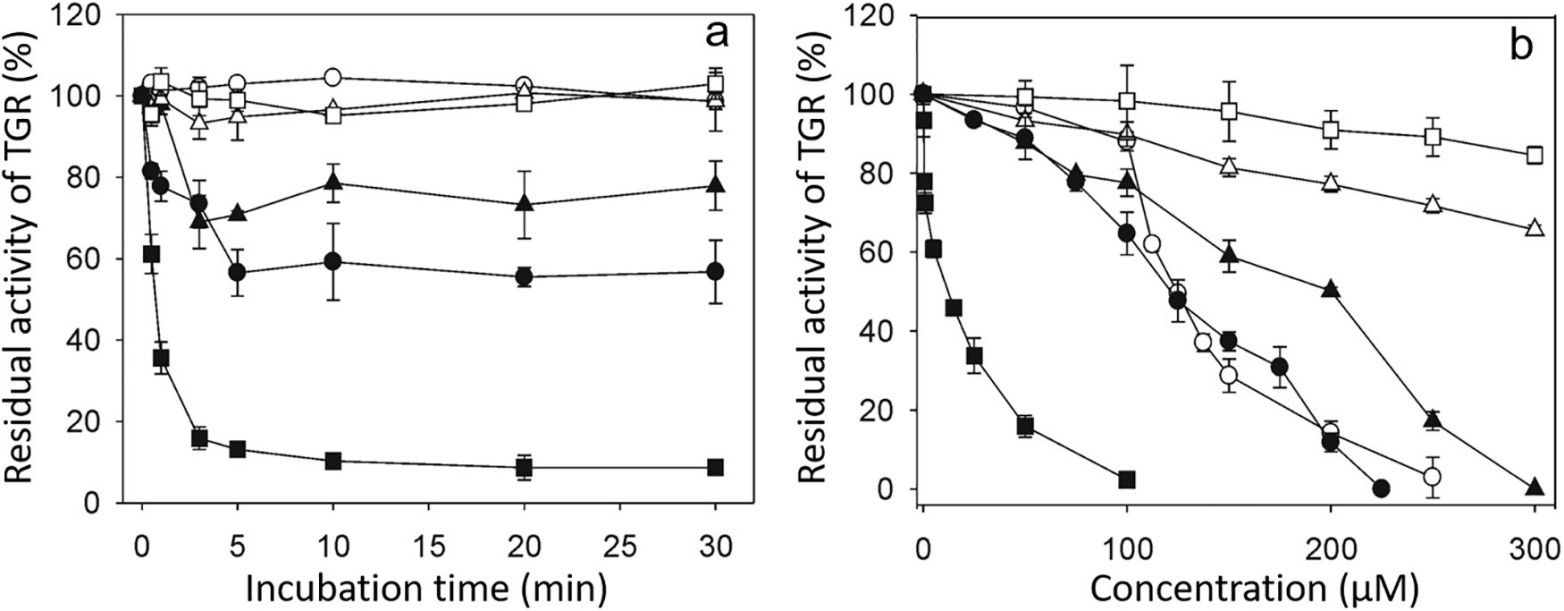
The inhibitory ability of curcumin degradation products (CDP) on the GSSG reductase activity of *Tc*TGR. (a). An enzyme aliquot was incubated in TE buffer in the presence of the corresponding CDP at the indicated concentration and 100 μM NADPH. After 3 min, the reaction was started by adding GSSG 67 μM. (b). An enzyme aliquot (14 nM) was incubated in TE buffer in the presence of 50 μM of the corresponding CDP. At the indicated times, the reaction was started by addition of 67 μM GSSG. For both experiments, the initial velocity determined in the absence of any curcumin derivative was taken as 100% enzyme activity. (□), vanillinic acid; (Δ), ferulic acid; (o), vanillin; (▲), feruloylmethane; (●), ferulic aldehyde; (■) 4-vinylguaiacol. When the enzyme was incubated in the presence of a constant concentration (50 μM) of the corresponding compound for a variable length of time, a similar inhibitory ability was observed.

To gain insight into the potential inhibitory ability of the curcumin breakage products feruloylmethane (FM), feruloyl aldehyde (F-Ad), 4-vinylguaiacol (4VG), vanillinic acid (VA), and ferulic acid (FA), its effect on the GSSG reductase activity of *Tc*TGR was tested. As shown in Fig 6a, the inhibitory ability of such curcumin derivatives was very different, ranging from a powerful inhibitor such as 4-vinylguaiacol (IC_50_ = 12.9 μM) to compounds with poor inhibitory ability, including ferulic acid (IC_50_ = 465 μM) and vanillinic acid (IC_50_ = 650 μM).

### Docking analysis between curcumin and curcumin derivates and thioredoxin glutathione reductase of *T*. *solium* (*Ts*TGR)

The superposition of the model obtained from *Ts*TGR with *Eg*TGR (PDB 5W1) is shown in S1 Fig; the RMSD between both structures is 0.254 Å. The coverage and identity of the amino acid sequence are 98 and 90%, respectively. The residues involved in the catalysis and binding of ligands are conserved, such is the case of Cys^156^ and Cys^161^ in *Eg*TGR (Cys^150^ and Cys^155^ in *Ts*TGR), and both cestodes are closely related with *T*. *crassiceps*. Therefore, we can establish that our obtained model is reliable, and we can use it to carry out docking studies with the different ligands that we explore in this study.

**Fig 7:**
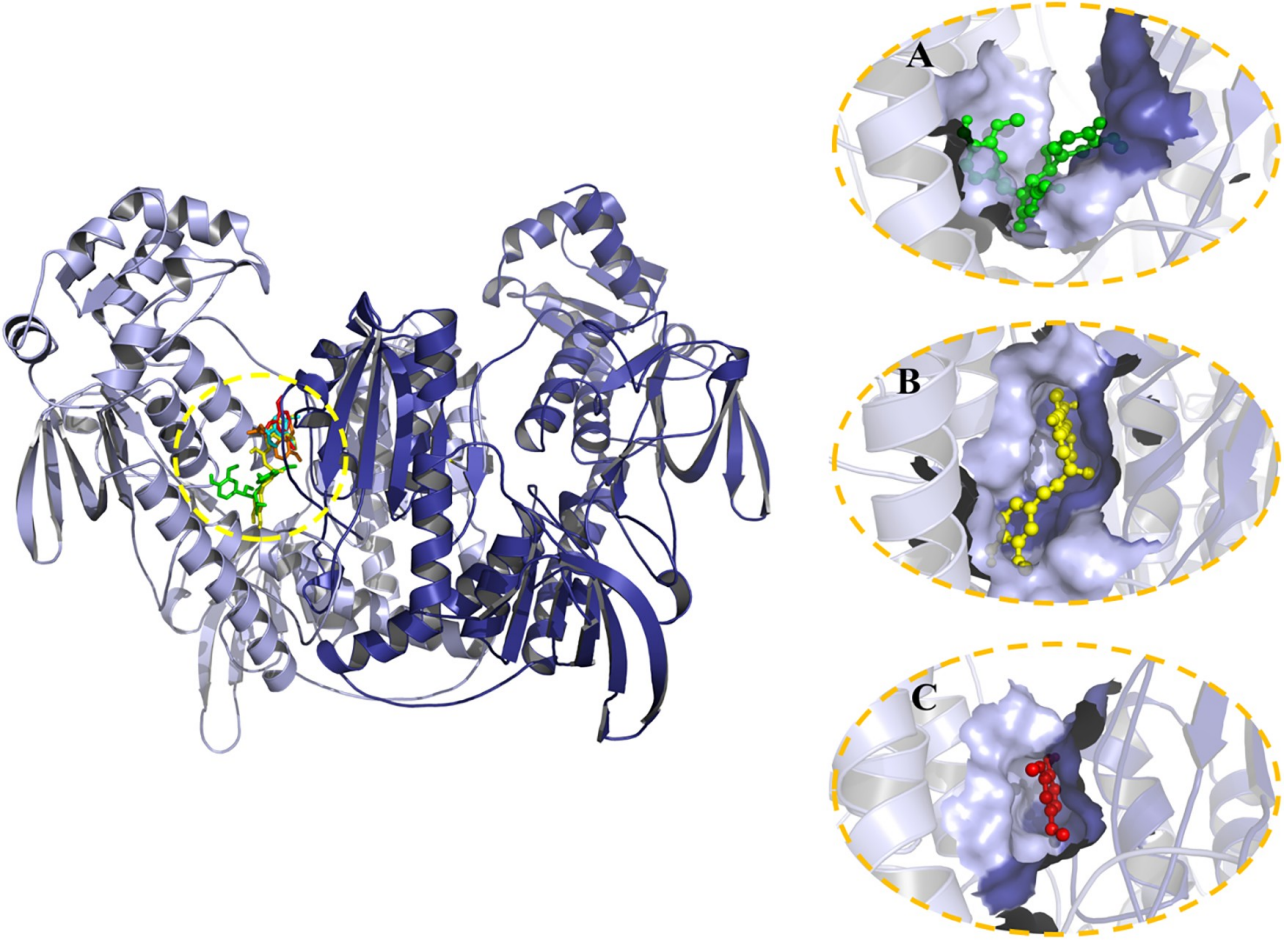
The predicted binding site of selected compounds on TGR from *T*. *solium*. The three-dimensional model of dimeric TGR from *T*. *solium* obtained from the structural modeling is shown at left. Encircled is the region of the protein at which both curcumin and curcumin derived compounds are bound as predicted by the docking analysis. The specific binding site for spiroepoxide (green sticks), curcumin (yellow sticks), or 4-VG (red sticks) is shown in expanded view at right.

**Fig 8:**
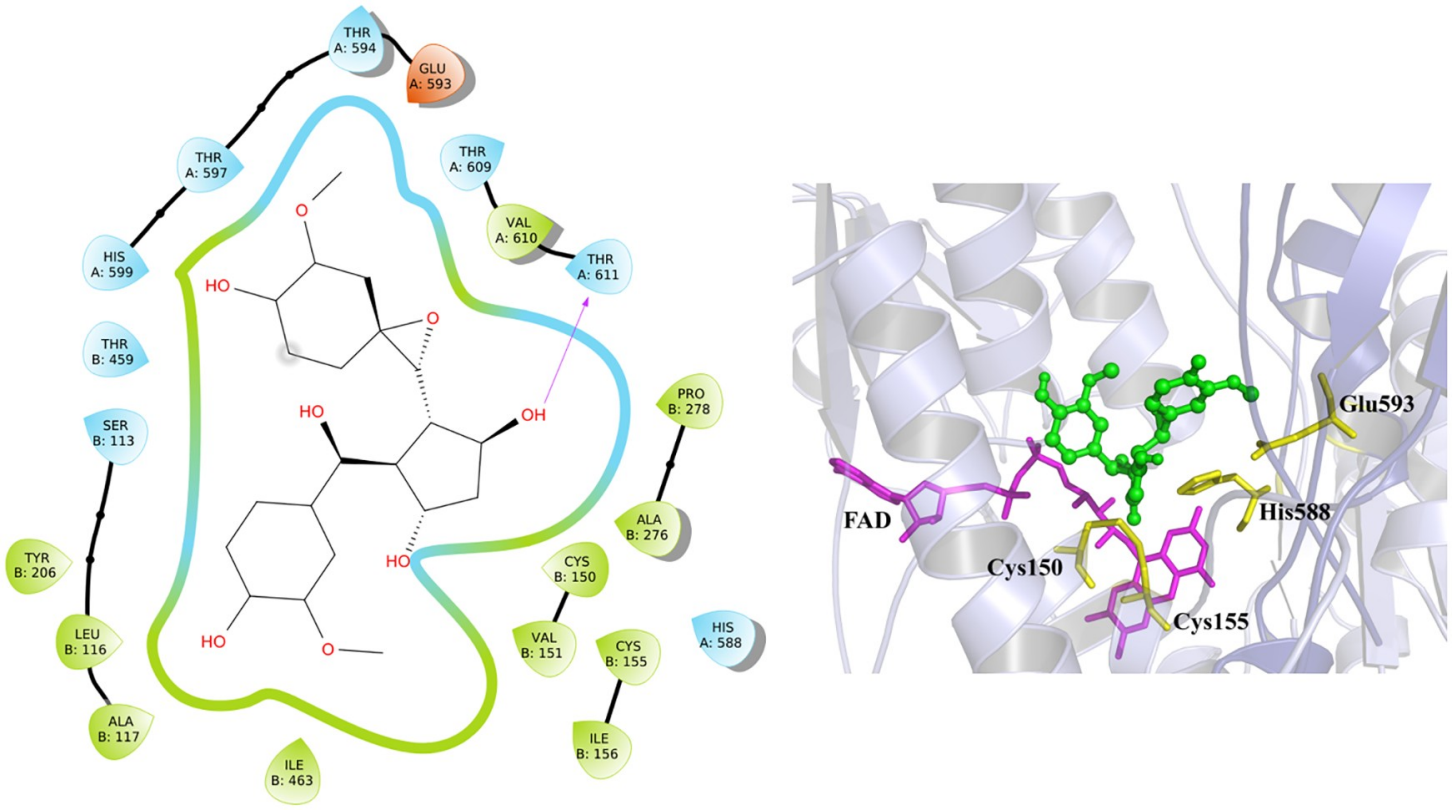
Binding site and interactions of spiroepoxide on TGR. Scheme (a) shows a two-dimensional view of the amino acid residues of the enzyme involved in the interaction with spiroepoxide. In (b) a three-dimensional view of the binding site showing FAD (purple sticks), spiroepoxide (green sticks), as well as the catalytically essential Cys^150^, Cys^155^, His^588^, and Glu^593^ (yellow sticks) is shown.

Docking studies were carried out using the *Ts*TGR model and seven compounds: curcumin, spiroepoxide, DMC, BDMC, VA, FA, and 4VG. The results obtained showed that all the compounds join in the region of the enzyme interface, which corresponds to the catalytic site of the same. Curcumin and spiropoxide demonstrate practically overlapping binding regions for the compounds (Fig 7), while 4VG, VA, and FA exhibit a different site (S2 Fig). The inhibition constants (*K*_i_) estimated by AutoDock4 for the compounds are DMC 0.37, BDMC 0.50, curcumin 0.63, spiroepoxide 1.77, 4VG 42.46, VA 61.54, and FA 68.69 μM. The compounds with the highest affinity correspond to the largest and they overlap at the same binding site. On the other hand, smaller compounds have less affinity for the enzyme, and they overlap each other sharing the same binding site. Experimentally, we observed that the product of oxidative degradation of curcumin, in this case, the spiroepoxide presents a greater degree of inhibition of the TGR in comparison with the others. Analyzing in detail the interaction of the TGR-spiroepoxide complex (Fig 8); we observed that spiroepoxide is bound in the catalytic site of the enzyme interacting directly with the catalytic residues (Glu^593^, His^588^, Cys^155^, and Cys^150^) and the FAD, which could interfere with the normal flow of electrons and inactivate the enzyme.

## Discussion

It has been suggested that the multiple pharmacological effects of curcumin are due to its derivatives obtained either by breakage or by oxidative modifications [11, 47] such proposal is based on both the chemical instability of curcumin in aqueous media, as well as the low concentrations at which the compound was found in plasma [7, 11]. On the other hand, from the results reported in the literature on the effect of the curcuminoids on enzyme activity [48–52], it has become clear that the presence of oxidant conditions is imperative in order to observe inhibition, particularly with curcumin. Thus, although xanthine oxidase was reported to be inhibited by curcumin [53], a degradation product (trans-6-{4’-hydroxy-3’-methoxyphenyl-2,4-dioxo-5-hexenal) appears to be involved in the inhibitory effect [54]. In a similar sense, the inhibitory ability of curcumin on human type II topoisomerase was dependent on the presence of an oxidant compound in the reaction mixture [55]. The effect of curcumin on recombinant TrxR from rat was reported [32], however, in that work no oxidative compound was used, and long incubation times in the presence of the curcuminoid were necessary in order to observe inhibition. Furthermore, the latter is reported as irreversible.

The present work sought to explore the inhibitory effects of curcuminoids, with the knowledge that they are unstable compounds when present in aqueous solutions, particularly curcumin. The oxidative modifications represent the major pathway by which curcuminoids are converted into a variety of compounds. Recently, a detailed mechanistic pathway for the production of a variety of derived compounds during curcumin autoxidation was proposed [45]. In the final step of the oxidative modifications, the stable product bicyclopentadione is produced. In such a process the concentration of all the intermediaries is time-dependent. Particularly noticeable in this sense was the appearance of an intermediary compound with a clear absorbance of ultraviolet light, a dioxygenated derivative of curcumin, which was demonstrated to correspond to the spiroepoxide intermediary. The compound has an absorption band with a maximum at 263 nm. The maximal abundance of the latter was reached at about 10 min after dilution [45]. As shown in S3 Fig, a chromatogram on RP-HPLC of a sample with curcumin in aqueous solution for 15 min revealed the presence of a compound with m/z of 399. This mass coincides with the expected molecular mass for the dioxygenated-curcumin derivate “spiroepoxide”, which in addition has an absorption signal at 263 nm equal to that shown in Fig 3. However, due to the great instability of this compound, we have not advanced in its characterization.

Interestingly, the susceptibility of curcumin to oxidative modifications were reported to be in sharp contrast with that of the related DMC and BDMC [10]. For DMC, the final product of their oxidative transformation was achieved 48 h after dilution, while for BDMC the addition of an oxidant compound (e.g. potassium ferrocyanide, K_3_Fe(CN)_6_ was required. In contrast, under our measurement conditions, both DMC and BDMC (99%, purity) are susceptible to undergo precipitation, as shown in S4 Fig. In this test, the absorption at 426 nm of the three curcuminoids was followed for several minutes. When the absorption decreased, the precipitate was resuspended in DMSO at the initial volume and the absorption was again measured. BDMC precipitate faster than DMC and only a small percentage of curcumin precipitates.

The above result suggests that the inhibition of the TGR activity by BDMC (Fig 1), could be apparent since the decrease in absorbance at 340 nm occurs by both NADPH oxidation and precipitation of the BDMC.

The results obtained in the present work suggest curcumin is not directly involved in the inhibition of *Tc*TGR. Instead, a short-lived curcumin derived compound produced by the oxidative pathway is the most likely to be the inhibitory molecule. Thus, preincubation of the enzyme at short time points in the presence of increasing concentrations of curcumin resulted in no inhibition, even at relatively high concentrations (Fig 1a). The long incubation time (in the range of minutes) needed to observe inhibition of *Tc*TGR by curcumin is consistent with the gradual accumulation of a curcumin derived compound. The time point at which the enzyme is fully inhibited is coincident with the maximal value of the absorbance at 263 nm. The results shown in Fig 4 strongly suggest the putative inhibitor has a short-lived existence that is not present when for the assay for TGR activity with a sample of oxidized curcumin during 45 min was used. It is worth to note that in spite of a very similar structure and a nearly identical *K*_d_ value, the related curcuminoids have a different inhibitory effect on TGR.

Taking into account the relatively high affinity of *Tc*TGR for curcumin revealed by the docking analysis (*K*_d_ = 0.63 μM), as well as the concentration of the compound used in the enzyme assays (50 μM), it would be expected that curcumin itself would be a suitable inhibitor. However, this is not the case. Such apparent contradiction can be explained by assuming the early stages in the oxidative pathway of curcumin are very fast, thus leading to an abrupt fall in the concentration of the compound after a few seconds after dilution in the assay buffer. The participation of reactive radical species in such early stages is consistent with such a proposal. In addition, docking simulation of *Ts*TGR with spiroepoxide demonstrates that the binding site is in the vicinity of the Cys^150^ and Cys^155^ (Fig 8) of the first catalytic site interrupting the electron transfer to the selenocysteine residue of the neighboring enzyme subunit, suggesting the TGR-inhibition mechanism. This is sustained for protection assay of low weight molecular thiols with the curcumin-oxidation products where the addition of exogenous thiols avoid TGR inhibition (Table 1).

On the other hand, the apparent stabilizing effect that NADPH has on curcumin provides clues into the mechanism of its destabilization. Thus, in the presence of the nicotinamide nucleotide, the oxidative modifications of curcumin are delayed, resulting in no inhibition of the enzyme. In this sense, even after four hours incubation in the presence of NADPH, no increase in the absorbance at 263 nm was observed, coincident with a total lack of inhibition. In this sense, it has been reported that curcumin reduction or conjugation gives less active or inactive metabolites [11] [56]. In the proposed oxidative pathway of curcumin [45] a branching reaction leading to the reduction of a radical endoperoxide intermediary is described. It is possible that in the presence of NADPH such a reaction could successfully to compete with the formation of this endoperoxide intermediary, leading to the formation of spiroepoxide, thus explaining the absence of inhibition when curcumin was preincubated with the reduced nicotinamide nucleotide. With respect to the protective effect of sulfhydryl compounds on the curcumin-dependent inhibition of TGR, it has been demonstrated that in the presence of such compounds (e.g., glutathione, NAC, DTT), curcumin forms covalent adducts with electrophiles molecules such as spiroepoxide. Interestingly, the thiol adducts of the spiroepoxide intermediary were the most abundant derivative compounds found [46]. Thus, the presence of the sulfhydryl compound leads to a significant reduction of spiroepoxide concentration, and hence no inhibition is observed. In this sense, when the thiol compound was added after 15 min of curcumin dilution, the observed protective effect was scarce (Fig 4).

In summary, based on the evidence available elsewhere, it is proposed the spiroepoxide derivative of curcumin oxidation is directly involved in the inhibitory effect of TGR. This conclusion is supported by the following experimental evidence: i) the time-dependent inhibitory effect of curcumin, which suggests a chemical transformation of the compound is needed in order to generate the real inhibitor; ii) the correspondence between the time of appearance of the maximal absorbance at 263 nm and the time at which the maximal inhibition was reached; iii) the ability of sulfhydryl reagents, which would block the oxidation curcumin derivative to prevent the inhibition of the enzyme; iv) the presence of molecule with m/z 399, which coincide with theoretical mass of spiroepoxide; and v) the existence of a potential binding site with high affinity for spiroepoxide (K_d_ = 1.63 μM), as derived from docking simulations.

The inhibitory effect of the CDP on *Tc*TGR demonstrated in the present work revealed a broad spectrum of effectivity (Fig 6), ranging from a very powerful inhibitor such as 4-vinylguaiacol up to compounds with a very poor inhibitory ability (e.g. ferulic acid) in spite of their structural resemblance. It is worth noting that for these kinds of curcumin derivatives no long incubation time was necessary in order to observe inhibition of the enzyme activity, demonstrating no chemical transformation is required for its inhibitory action. In this sense, analysis of the time dependence of the absorption spectrum of 4-vinylguaiacol, revealed high stability of the compound (S5 Fig). Results of the docking simulations with 4-vinylguaiacol revealed a putative binding site located at the subunit interface of the enzyme (S2 Fig). The higher *K*_d_ values obtained for this kind of curcumin derivatives, as compared with that of the curcuminoids, are consistent with a lesser surface area interacting with the enzyme.

The identification of the precise chemical nature of the compound(s) involved in the multiple biological effects reported for curcumin is a very important issue. In the present work, we have found that curcumin is not responsible for TGR inhibition. Instead, an autoxidation product of the compound that is directly involved in the observed inhibition. These results point to curcumin as a promising pro-drug to be used in fighting this kind of parasites.

## Supporting information

S1 Fig Overlapping of the three-dimensional structure of TGR from *E*. *granulosus* and *T*. *solium*. Superposition of *Ts*TGR (yellow-orange) and *Eg*TGR (light blue). The structures were overlaid via the backbones of the structures using program Pymol.
(TIF)

S2 Fig Three-dimensional models of the interactions between the *Ts*TGR and its ligands. A. Curcumin (yellow sticks), spiroepoxide (green sticks), DMC (purple-sticks), and BDMC (white sticks). B. Curcumin degradation products (CDP): 4-vinylguaiacol (red sticks), ferulic acid (orange sticks), and vanillin (cyan sticks).
(TIF)

S3 Fig ESI chromatogram of curcumin and its oxidation products. LC/MS analysis was performed using an Agilent Technologies (G6410 LCMS) triple stage quadrupole MS using electrospray ionization in the negative ion mode. Chromatographic separation of metabolites was achieved using an extend C-18 column (4.6 × 150 mm, 5 μm), eluted at a flow rate of 0.5 mL/min. Samples were eluted from the column using a linear gradient of 5–95% acetonitrile containing 0.1% formic acid over. Inset, LC-ESI mass spectra shows mainly *m/z* 367 and 399 that coincides with curcumin and deoxygenated curcumin respectively.
(TIF)

S4 Fig Precipitation of curcuminoids. A. A 50 μM solution of either curcumin, DMC, and BDMC was prepared in TE buffer in a final volume of 500 μL and its absorbance at 426 nm measured. B. After standing at room temperature, the absorbance of the solutions was again measured at the indicated times (in parenthesis). Then, the upper 450 μL of the corresponding solution was removed carefully. C. After the addition of 450 μL of DMSO to each solution, its absorbance at 426 was determined.
(TIF)

S5 Fig The absorption spectrum of curcumin degradation products (CDP). The corresponding compound was diluted to a final concentration of 50 μM in TE buffer and scanned between 230 to 500 nm. (A) 4-vinylguaiacol; (B) vanillic acid; (C) ferulic aldehyde; (D) ferulic acid; (E) feruloyl methane; (F) vanillin. In each case, the wavelengths of maximal absorbance are indicated.
(TIF)
