# Correction: Effect of curcuminoids and curcumin derivate products on thioredoxin-glutathione reductase from *Taenia crassiceps cysticerci*. Evidence suggesting a curcumin oxidation product as a suitable inhibitor

**DOI:** 10.1371/journal.pone.0223795

**Published:** 2019-10-07

**Authors:** Alberto Guevara-Flores, José de Jesús Martínez-González, Álvaro Miguel Herrera-Juárez, Juan Luis Rendón, Martín González-Andrade, Patricia Victoria Torres Durán, Raúl Guillermo Enríquez-Habib, Irene Patricia del Arenal Mena

There is an error in affiliation 2 for author Raúl Guillermo Enríquez-Habib. The correct affiliation 2 is: Departamento de Química Orgánica, Instituto de Química, Universidad Nacional Autónoma de México, Mexico City, Mexico.
