# Supplementary figures and images for: Effect of curcuminoids and curcumin derivate products on thioredoxin-glutathione reductase from Taenia crassiceps cysticerci. Evidence suggesting a curcumin oxidation product as a suitable inhibitor
Source: PLoS One. 2019 Jul 22;14(7):e0220098. doi: 10.1371/journal.pone.0220098 (PMC6645542; doi:10.1371/journal.pone.0220098)

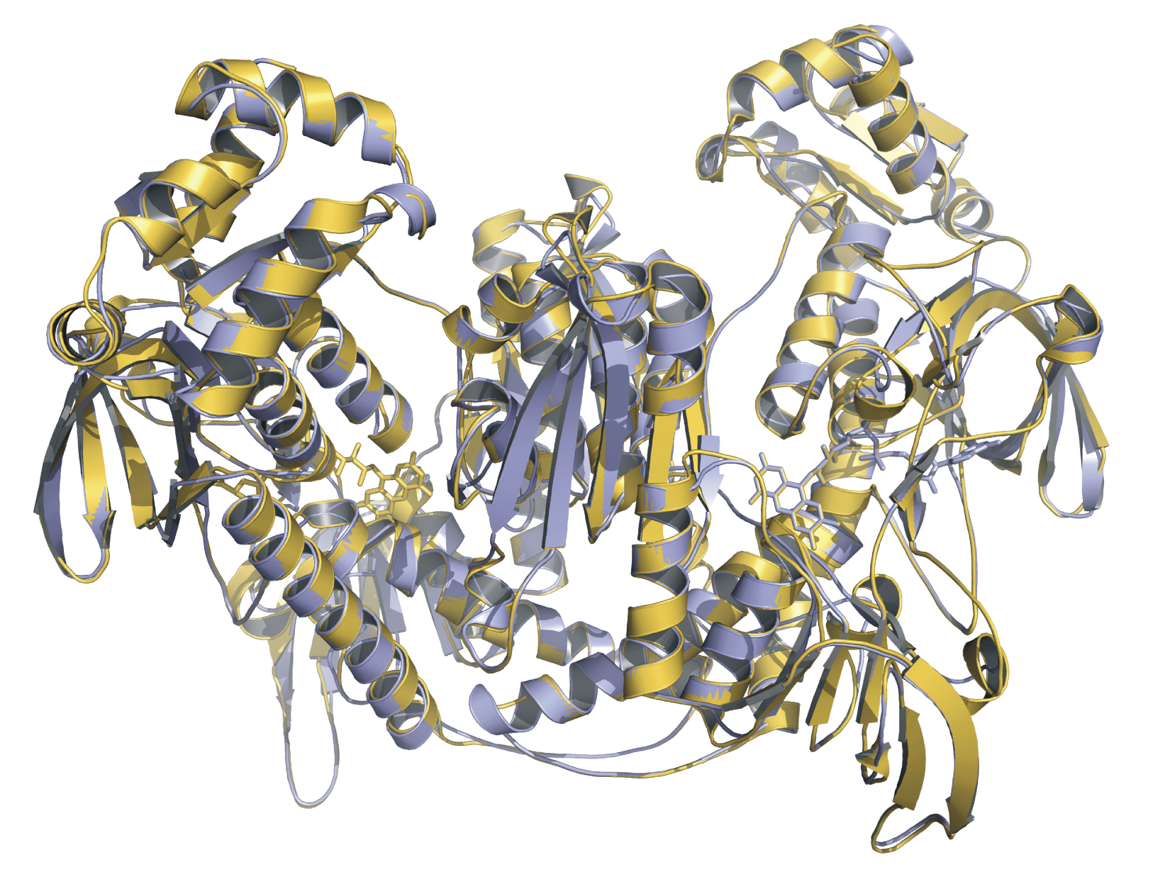

Supplement: S1 Fig — Superposition of TsTGR (yellow-orange) and EgTGR (light blue). The structures were overlaid via the backbones of the structures using program Pymol. (TIF) [file pone.0220098.s001.tif]

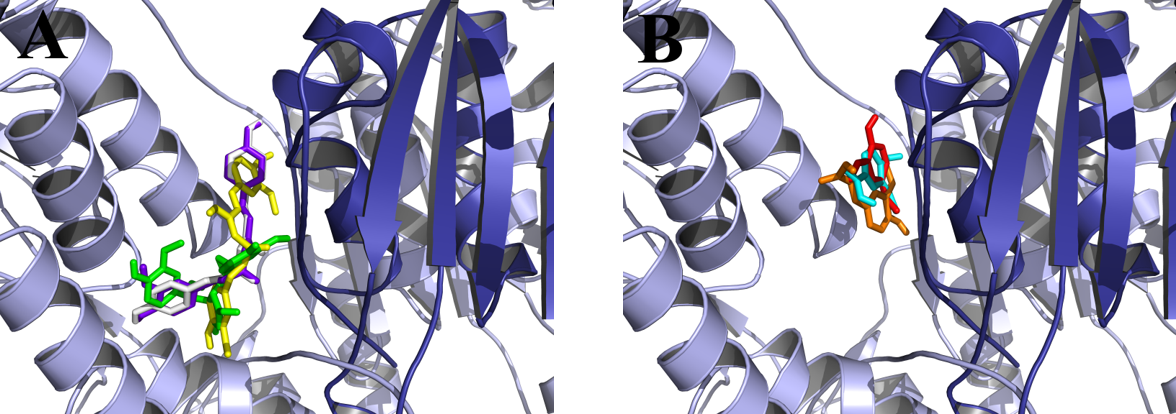

Supplement: S2 Fig — A. Curcumin (yellow sticks), spiroepoxide (green sticks), DMC (purple-sticks), and BDMC (white sticks). B. Curcumin degradation products (CDP): 4-vinylguaiacol (red sticks), ferulic acid (orange sticks), and vanillin (cyan sticks). (TIF) [file pone.0220098.s002.tif]

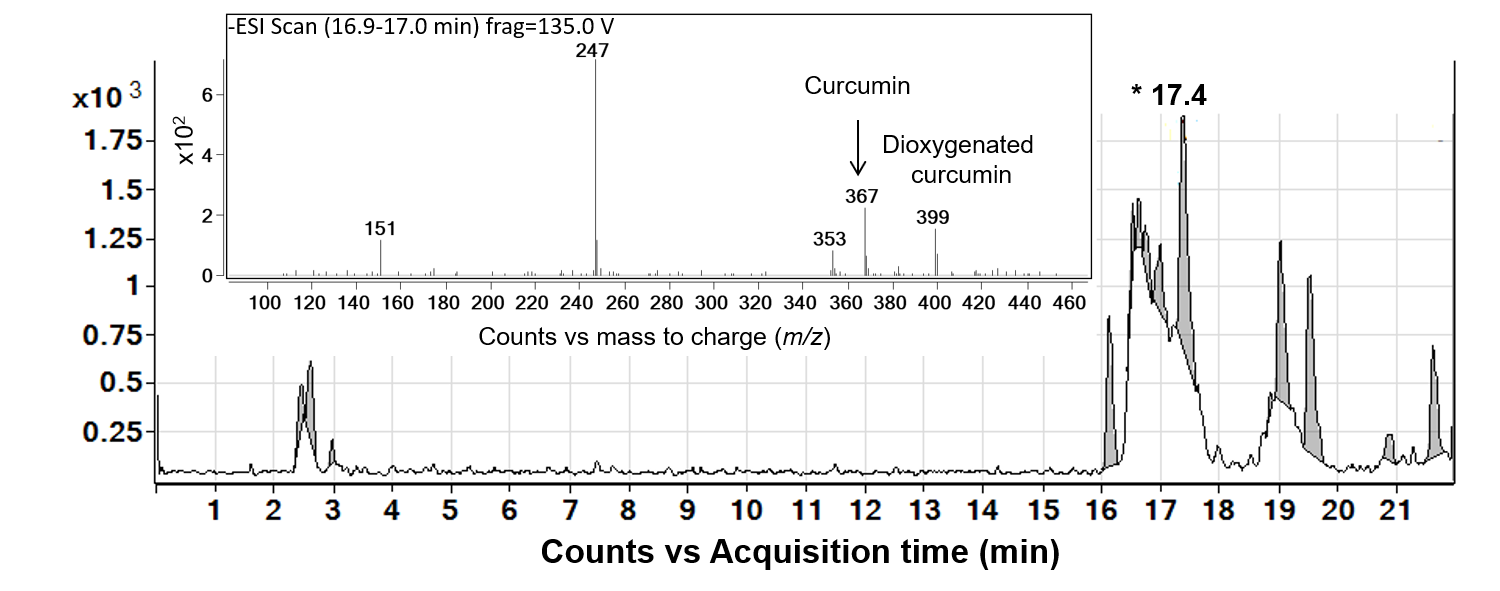

Supplement: S3 Fig — LC/MS analysis was performed using an Agilent Technologies (G6410 LCMS) triple stage quadrupole MS using electrospray ionization in the negative ion mode. Chromatographic separation of metabolites was achieved using an extend C-18 column (4.6 × 150 mm, 5 μm), eluted at a flow rate of 0.5 mL/min. Samples were eluted from the column using a linear gradient of 5–95% acetonitrile containing 0.1% formic acid over. Inset, LC-ESI mass spectra shows mainly m/z 367 and 399 that coincides with curcumin and deoxygenated curcumin respectively. (TIF) [file pone.0220098.s003.tif]

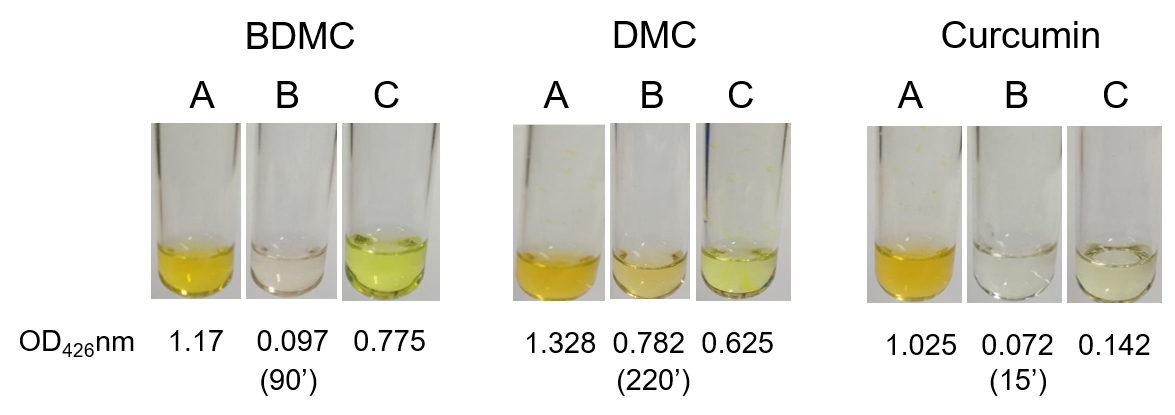

Supplement: S4 Fig — A. A 50 μM solution of either curcumin, DMC, and BDMC was prepared in TE buffer in a final volume of 500 μL and its absorbance at 426 nm measured. B. After standing at room temperature, the absorbance of the solutions was again measured at the indicated times (in parenthesis). Then, the upper 450 μL of the corresponding solution was removed carefully. C. After the addition of 450 μL of DMSO to each solution, its absorbance at 426 was determined. (TIF) [file pone.0220098.s004.tif]

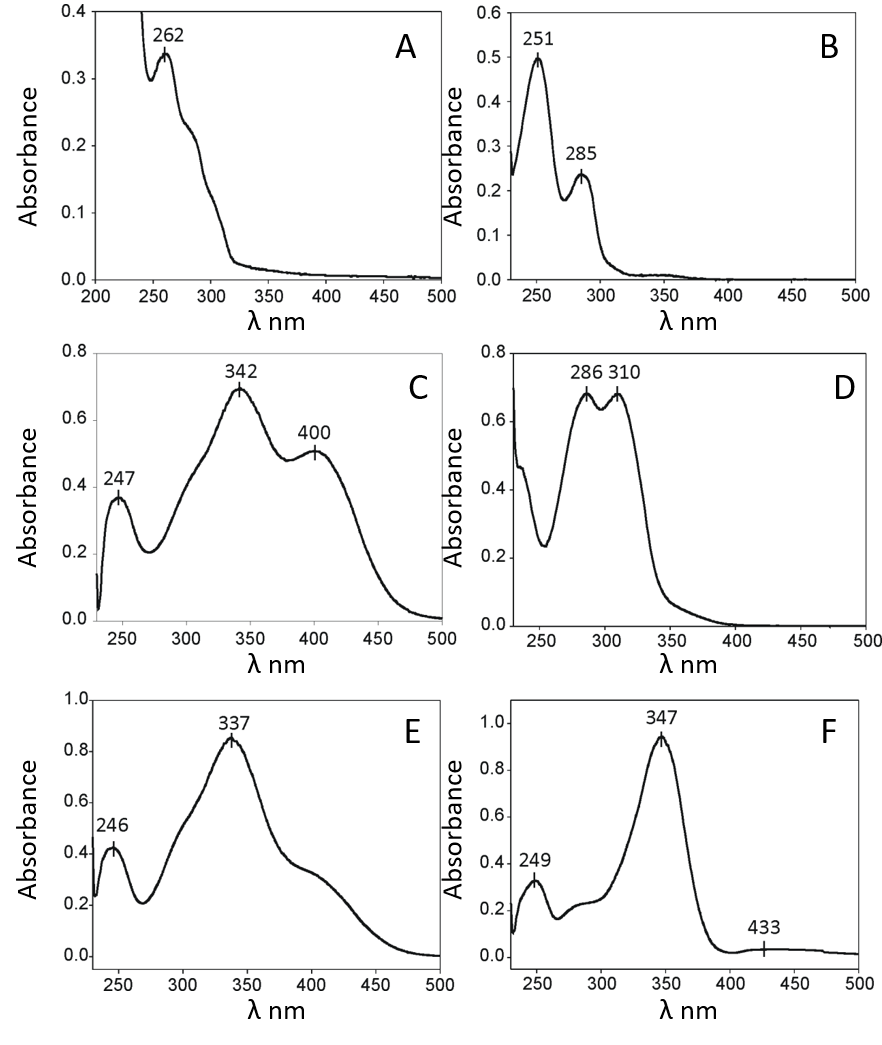

Supplement: S5 Fig — The corresponding compound was diluted to a final concentration of 50 μM in TE buffer and scanned between 230 to 500 nm. (A) 4-vinylguaiacol; (B) vanillic acid; (C) ferulic aldehyde; (D) ferulic acid; (E) feruloyl methane; (F) vanillin. In each case, the wavelengths of maximal absorbance are indicated. (TIF) [file pone.0220098.s005.tif]
